# Mothers’ Diet and Family Income Predict Daughters’ Healthy Eating

**DOI:** 10.5888/pcd18.200445

**Published:** 2021-03-18

**Authors:** Christopher D. Pfledderer, Lisa H. Gren, Julie Metos, Timothy A. Brusseau, Karen O’Toole, Saundra S. Buys, Mary B. Daly, Caren J. Frost

**Affiliations:** 1 Department of Health and Kinesiology, University of Utah, Salt Lake City, Utah; 2 Department of Family & Preventive Medicine, University of Utah, Salt Lake City, Utah; 3 Department of Nutrition and Integrative Physiology, University of Utah, Salt Lake City, Utah; 4 Huntsman Cancer Institute, University of Utah, Salt Lake City, Utah; 5 Fox Chase Cancer Center, University of Pennsylvania, Philadelphia, Pennsylvania; 6 College of Social Work, University of Utah, Salt Lake City, Utah

## Abstract

**Introduction:**

Understanding the degree to which parents may influence healthy behaviors may provide opportunities to intervene among populations at increased risk of diseases, such as breast cancer. In this study, we examined the association between daughters’ healthy eating habits and family lifestyle behaviors among girls and their families by using baseline data from the LEGACY (Lessons in Epidemiology and Genetics of Adult Cancer from Youth) Girls Study. Our objective was to examine the relationship between daughters’ healthy eating and family lifestyle behaviors and to compare these associations between families with and without a history of breast cancer.

**Methods:**

We examined demographic and lifestyle data from a cohort of 1,040 girls aged 6 to 13 years from year 1 (2011) of the LEGACY study. Half had a family history of breast cancer (BCFH). We used mixed-effects linear regression to assess the influence of the mother and father’s physical activity, family relationship scores, the mother’s diet, the family’s income, and the daughter’s sports participation, age, body mass index (BMI), and race/ethnicity on the daughter’s Healthy Eating Index (HEI) score.

**Results:**

Daughters’ healthy eating was significantly correlated with the mother’s diet (*r*[668] = 0.25, *P* = .003) and physical activity (*r*[970] = 0.12, *P* = .002), the father’s physical activity (*r*[970] = 0.08, *P* = .01), and the family income (*r*[854] = 0.13, *P* = .006). Additionally, the mother’s diet (β coefficient = 0.71, 95% CI, 0.46–0.88, *P* = .005) and family income (β coefficient = 3.28, 95% CI, 0.79–5.78, *P* = .002) significantly predicted a daughter’s healthy eating. Analyses separated by family history status revealed differences in these associations. In families without a history of breast cancer, only the mother’s diet (β coefficient = 0.62; 95% CI, 0.29–0.95; *P* = .001) significantly predicted the daughter’s healthy eating. In families with a history of breast cancer, the mother’s diet (β coefficient = 0.73, 95% CI, 0.42-1.03, *P* = .006) and family income (β coefficient = 6.24; 95% CI, 2.68–9.80; *P* = .004) significantly predicted a daughter’s healthy eating.

**Conclusion:**

A mother’s diet and family income are related to the daughter’s healthy eating habits, although differences exist among families by family history of breast cancer.

**Summary**

**What is already known on this topic?**

Diet and physical activity can promote adult health and are 2 modifiable lifestyle behaviors in youth that parents can influence. Understanding the degree to which parents may influence these behaviors may provide opportunities to intervene among populations at increased risk for breast cancer.

**What is added by this report?**

Findings from this study reinforce the importance of parental influence on daughters’ eating habits and the value of a family-focused approach to addressing breast cancer prevention and overall health.

**What are the implications for public health practice?**

Our study contributes to the growing body of literature on the effect of family behavior on adolescent health. The study has several implications for how researchers, practitioners, and educators view health interventions aimed at girls and young women with and without a family history of breast cancer.

## Introduction

Incidence of breast cancer, the most common cancer in women, is on the rise globally (1). Adult daughters in families with a history of the disease are at greater risk of breast cancer than women without a family history (2). Increasing evidence suggests that early lifestyle behaviors can affect a woman’s breast cancer risk (3). Children and adolescents may be particularly sensitive to exposures associated with breast cancer initiation or that protect against breast cancer development. Consequently, a current focus is on how early lifestyle modifications (in adolescence) may reduce a woman’s risk of breast cancer later in life (4). Although family history is not modifiable (5), lifestyle behaviors such as physical activity and diet can be changed to reduce the risk of diseases such as breast cancer (6,7). Evidence supports an inverse relationship between physical activity and breast cancer and a positive relationship between obesity and breast cancer (8). Although no dietary components are associated with breast cancer, the consistent relationship between obesity and breast cancer and other health conditions warrants assessment of overall diet in youth.

Diet and physical activity can promote adult health and are 2 modifiable lifestyle behaviors in youth that parents can influence (6). Social influences at mealtime affect both food quantity and preference. Families that eat together tend to eat more healthful foods and regulate the amount eaten (9–11). This is often called a modeling effect. In Bandura’s *Social Learning Theory*, modeling is described as a powerful way to adopt behaviors through watching and imitating others, such as parents and siblings (12). Modeling may also influence physical activity, particularly between girls and their parents and in children’s participation in organized sports (13–15). Furthermore, parents create social norms around eating and physical activity through the food and exercise opportunities they provide and the attitudes they project about healthful behaviors (16). Norms are especially influential because people by nature observe and mimic behavior and take pleasure from being rewarded for their behaviors (17). Parenting styles also influence adoption of lifestyle behaviors; encouragement and boundary setting promote healthful behaviors, and coercion is aversive (11,17,18). However, research on parental influence has yielded mixed results. Some studies show that parents’ diet and physical activity are co-connected and are linked to and promote the health of their children (6,19). Other studies do not show this effect (20). Vepsäläinen et al (21) found that adolescent diets are more closely linked to the parent who reported the food consumed and may not accurately reflect what children are actually eating. In a 2016 study, Kwon et al (6) found that the father’s involvement meant that children aged 5 to 19 years were more likely to engage in sustained sports activities. Niermann et al (19) found that physical activity levels and healthy diets were an issue for the family as a whole. Other research showed that when mothers modified their physical activity and diet, daughters had healthier diets and better physical activity habits (22).

Aside from diet and physical activity, numerous other family characteristics and lifestyle behaviors may affect the health of children. For example, a systematic review of the relationship between family functioning and adolescent overweight and obesity demonstrated that family functioning is significantly related to obesity (23). In this review, family functioning was defined as the interactions between family members, which makes it a modifiable behavior. Additionally, Berge et al (24) found that for adolescent girls, high family functioning was significantly associated with less sedentary behavior and a high intake of fruits and vegetables, which provides evidence for underlying linkages between family dynamics and modifiable health behaviors, such as diet and physical activity.

Studies exploring parental influence on children’s lifestyle behaviors have yielded mixed results. However, understanding the degree to which parents may influence these behaviors may provide opportunities to intervene among populations at increased risk of diseases such as breast cancer. Our objective was to examine the associations between daughters’ healthy eating and family lifestyle behaviors and to compare these associations between families with and without a history of breast cancer. 

## Methods

### Study design

Data for our study were collected for the LEGACY (Lessons in Epidemiology and Genetics of Adult Cancer from Youth) Girls Study, a cohort study begun in 2011. Girls, aged 6 to 13 years at the time of enrollment, and their mothers were enrolled at 5 sites in North America: Toronto, Canada; San Francisco, California; New York, New York; Philadelphia, Pennsylvania; and Salt Lake City, Utah. Briefly, mothers who were part of the Breast Cancer Family Registry (http://www.bcfamilyregistry.org/) were contacted to ask whether they and their age-eligible daughters would participate in the study. In turn, mothers identified friends with age-eligible daughters who might also want to participate, and these women were contacted by study site personnel. Women and their daughters from the registry and their friends and daughters with a history of breast cancer in a first- or second-degree relative were classified as positive for breast cancer family history (BCFH positive). Those without such a history were classified as BCFH negative. No exclusion criteria beyond the daughter’s age were applied. Detailed methods and other study-related information are available elsewhere (25). The study, funded by the National Cancer Institute (2011–2016), considered the effect of behavior, environment, and diet on growth and development of girls from families positive and negative for breast cancer history.

Data for our analysis were collected in year 1 of the LEGACY study from girls and their mothers; girls aged 10 years or older completed questionnaires themselves, and girls aged 6 to 9 years completed questionnaires with help from their mothers. Data from multiple questionnaires were collected electronically and by paper surveys. Anthropometric measurements (height, weight, foot size, percentage body fat, and waist and hip circumference) were collected every 6 months. All data were entered into a Qualtrics (Qualtrics XM) database for analyses by researchers at the study sites and elsewhere. Our study considered baseline data only. This study was reviewed and approved by institutional review boards at each of the 5 participating sites: the University of Utah, Stanford University, Columbia University, Temple University, and the University of Toronto.

### Measures and data processing

**Demographic characteristics**. All demographic characteristics were self-reported by participating parents and consisted of daughter’s age and race/ethnicity, father’s education level, mother’s education level, family income range, and housing type.

**Diet**. The daughter’s Healthy Eating Index (HEI) score was our primary outcome of interest and was calculated by using methods recommended by the National Cancer Institute Dietary Assessment Primer (26). HEI-2010 measures diet quality and conformance with federal dietary guidelines. It includes 12 components that assess adequacy of the diet and dietary components that should be consumed in moderation. Final scores for HEI range from 0 to 100 and the higher the score, the higher the diet’s quality. HEI-2010 has been shown to be both a reliable and valid measure of diet quality in Americans aged 2 years and older and is widely used in child and adolescent dietary research (27). First, daughters’ responses to the Block Questionnaire for Ages 8–17 — 2004 Food Frequency Questionnaire (28) were analyzed, and results were translated into the HEI by using SAS (SAS Institute, Inc) code and statistical analysis methods described by the National Cancer Institute (29). Mothers’ diets were assessed with a set of 7 questions from the Block Food Frequency Questionnaire for adults that asked about eating habits from the past week (30,31). A typical question was “During the past 7 days, how many times did you eat green salad or other vegetables?” Respondents were asked to choose answers that ranged from “I did not eat green salad during the past 7 days” (coded as 0) to “4 or more times per day” (coded as 6). Items representing unhealthy eating habits including, “During the past 7 days, how many times did you eat sweets?” were reverse coded. The final total score ranged from 0 to 42, and the higher the score, the healthier the diet.

**Physical activity**. Daughters’ physical activity was assessed with a parent-reported questionnaire item that asked, “Has your daughter ever participated at least once a week for at least 1 season in any of the following organized activities?” followed by “How many minutes per week did she do the activity?” Parents were then asked to provide an answer in minutes per week of activities such as dance, soccer, basketball, softball, and ballet. Parent-reported minutes per week for each activity were totaled to create the final physical activity variable for daughters. Mothers’ physical activity was assessed with a self-reported questionnaire item that asked them to list the types of sports and exercise they do, followed by the number of minutes per week they engaged in that activity. Mothers reported fathers’ physical activity by responding to the same questions they were asked about themselves. The minutes per week of each activity were totaled to create the final physical activity variables for mothers and fathers.

**Anthropometric measures**. Participating daughters came to LEGACY offices at each site and were weighed and measured for height. Height (to the nearest half-centimeter) and weight (to the tenth of a kilogram) were measured twice at each visit by using the same equipment each time. If the 2 measures differed during the same visit, a third measurement was made to confirm the correct measure. Clinical tools used were the Omron Fat Loss Monitor with Scale (Model HBF-400) for weight and a standard stadiometer (Harpenden Pocket Stadiometer) for height (measured in bare feet). Body mass index (BMI) (weight in kg/height in m^2^) was calculated for each girl at every visit.

**Family relationships**. Family relationship scores were calculated by using the General Family Functioning (FAD-12) subsection of the McMaster Family Assessment Device (FAD) (32). The full measure comprises 60 statements about family structural, organizational, and transactional characteristics. Respondents rate how well each statement describes their family on a 1 to 4 Likert scale, ranging from “strongly agree” to “strongly disagree.” FAD-12 is a condensed version of the full measure and can be used as a stand-alone tool. A typical question from FAD-12 is a yes/no answer to “In times of crisis we can turn to each other for support.” Scoring of FAD-12 involves adding each respondent’s score and dividing by 12 for a final total score that ranges from 1 to 4. The higher the score, the more problematic the family’s functioning. Both mothers and daughters completed the FAD-12 separately, resulting in a unique score for each family member. FAD has been shown to be both a reliable and valid measure (33).

### Statistical analysis

We included LEGACY baseline data from 1,040 girls and their parent or guardian in our study. Data were screened for outliers by using boxplots and *z* scores and checked for Gaussian distributions by using a kernel density plot, which is a plot that visualizes the distribution of data over a continuous interval or time period. Descriptive statistics of participants were summarized by using sample percentages or means and standard deviations. Potential differences between BCFH-negative and BCFH-positive families were examined by using *t* tests for continuous variables and χ^2^ tests for categorical variables.

Potential correlates of daughters’ healthy eating scores were selected a priori to test their association with HEI scores on the basis of previous associations reported in the literature (6). Correlates included mothers’ self-reported diet, physical activity, family relationship scores, and fathers’ physical activity. Exploratory analyses of potential correlates were completed for daughters’ sports participation, age, BMI, race/ethnicity, and family income. We used bivariate Pearson correlations to identify any linear relationships between these selected variables and to screen preliminarily for multicollinearity among potential predictors to be used in subsequent linear regression models.

We examined the predictive utility of select family characteristics, including mothers’ and fathers’ physical activity, family relationship scores, mothers’ diets, family income, and daughters’ sports participation, age, BMI, and race/ethnicity with daughters’ HEI scores by using mixed-effects linear regression models with testing site as the nesting variable. This accounted for differences in sampling rates that occurred at each test site. Model 1 contained only family lifestyle characteristics: mothers’ and fathers’ physical activity, family relationship scores, mothers’ diets, and daughters’ sports participation. Model 2 contained all variables from Model 1 with the addition of select covariates. The covariates were daughters’ age and race/ethnicity, and family income. Data from 522 participants were included in the final linear regression model because of missing data from the total sample. The same analysis was conducted separately for BCFH-negative and BCFH-positive families. We computed tolerance and variance inflation factor (VIF) scores to ensure that no multicollinearity was present among predictors, with a tolerance value of <0.1 and a VIF score of >5.0 indicating the presence of multicollinearity among predictors in the current model.

Because more than half of the total sample was missing because of listwise deletion, we made statistical comparisons of the analytic subset and the subset of data that was removed because of missingness (Table 1). This revealed significant differences between the data sets for 2 variables, daughter’s BMI (*P* = .02) and daughter’s age (*P* = .004). However, these were not considered clinically significant differences. We also attempted multiple imputation by using Markov Chain Monte Carlo procedures to account for the large portion of missing data in the final analytic sample. However, no variables were found to adequately predict missingness, and the resulting regression models had estimates similar to the original regression models. All analyses had an initial α level of *P* < .05 and were calculated by using Stata version 16.0 (Stata Corp LLC).

**Table 1:** Comparison of Characteristics of the Analytic Subset and the Subset of Data Removed Because of Missingness, LEGACY Girls Study, 2011^a^

Characteristic	Analytic Subset (n = 522)	Removed (n = 618)	*P* Value^b^
**Daughters’ sports participation, min/wk, mean (SD)**	483.12 (965.01)	441.42 (1042.12)	.48
**Mothers’/guardians’ diet score^c^, mean (SD)**	31.63 (3.78)	31.50 (3.76)	.57
**Daughters’ HEI score^d^, mean (SD)**	62.24 (9.65)	61.89 (9.56)	.55
**Mother’s physical activity (min/week), mean (SD)**	212.05 (194.66)	204.20 (212.93)	.52
**Fathers’ physical activity (min/week), mean (SD)**	148.02 (195.62)	146.44 (278.15)	.89
**Family relationship score, mean (SD)^e^**	2.45 (0.01)	2.45 (0.07)	.22
**Daughters’ body mass index^f^, mean (SD)**	17.60 (3.54)	17.11 (3.58)	.02
**Daughters’ age, mean (SD)**	9.66 (2.29)	8.71 (2.19)	.004
**Daughters’ race/ethnicity, n (%)**			
Hispanic White	69 (13.3)	99 (16.0)	.07
Hispanic Black	9 (1.7)	16 (2.6)	
White	333 (64.2)	313 (50.6)	
African American/Black	41 (7.9)	37 (6.0)	
Native Hawaiian/Other Pacific Islander	2 (0.4)	1 (0.1)	
Asian	52 (9.6)	41 (6.6)	
Mixed race/ethnicity	16 (2.9)	11 (1.8)	
Missing	—	100 (16.2)	
**Annual income, n (%), $**			
<50,000	86 (16.6)	66 (10.7)	.85
50,000–74,999	52 (9.8)	48 (7.8)	
75,000–99,999	75 (14.1)	55 (8.9)	
≥100,000	309 (59.5)	261 (42.2)	
Missing	—	188 (30.4)	

Abbreviations: —, data missing; HEI, Healthy Eating Index.

a Lessons in Epidemiology and Genetics of Adult Cancer from Youth (25). Only variables included in the full linear regression model were used to compare the analytic sample and the removed sample.

b *P* values calculated by *t *tests for continuous variables and χ^2^ tests for categorical variables. Significant at *P* < .05.

c Out of 42; a higher number indicates a healthier diet score.

d Out of 100; a higher number indicates a healthier diet score.

e Out of 4; a higher relationship score indicates a healthier family relationship.

f Calculated as weight in kg/height in m^2^.

## Results

**Descriptive statistics**. Of the 1,040 girls in our study, 456 (43.8%) were BCFH negative (Table 2). Significant differences between BCFH negative and BCFH positive families existed for the variables of race/ethnicity (χ^2^[6, N = 1,044] = 29.68, *P* < .001), fathers’ education (χ^2^[6, N = 997] = 23.78, *P* = .002), income (χ^2^[5, N = 920] = 11.17, *P* = .048), and housing type (χ^2^[3, N = 961] = 13.50, *P* = .04). No significant differences (*P* < .05) between BCFH negative and BCFH positive families existed for any lifestyle variables (Table 3).

**Table 2:** Demographic Characteristics of Participating Families, by Breast Cancer Family History Status, LEGACY Girls Study, 2011^a^

Characteristic	All Girls, N = 1,040	BCFH Negative^b^, n = 456	BCFH Positive^b^, n = 584	*P* Value^c^
**Age, mean (SD)**	9.63 (2.36)	9.48 (2.22)	9.75 (2.46)	.07
**Race/ethnicity^d^**				
Hispanic White	168 (16.2)	76 (16.6)	92 (16.0)	<.001
Hispanic Black	25 (2.4)	10 (2.2)	15 (2.6)	
White	646 (62.1)	249 (54.3)	397 (67.9)	
African American/Black	78 (7.5)	48 (10.6)	30 (5.1)	
Native Hawaiian/Other Pacific Islander	3 (0.3)	2 (0.4)	1 (0.2)	
Asian	93 (8.9)	53 (11.6)	40 (6.7)	
Mixed race/ethnicity	27 (2.6)	18 (3.9)	9 (1.4)	
**Fathers’ education^d^**				
≤High school graduate	145 (13.9)	63 (13.5)	82 (14.3)	.002
Some college or university	152 (14.6)	67 (14.6)	85 (14.6)	
Bachelor’s degree	296 (28.4)	111 (24.6)	185 (31.4)	
Graduate degree	338 (32.5)	157 (34.6)	181 (30.9)	
Vocational/technical school	42 (4.0)	22 (4.7)	20 (3.5)	
Not reported	20 (2.0)	16 (3.6)	4 (0.8)	
Missing	47 (4.5)	20 (4.4)	27 (4.6)	
**Mothers’ education^d^**				
≤High school graduate	80 (7.7)	36 (8.0)	44 (7.6)	.83
Some college or university	171 (16.4)	73 (16.2)	98 (17.1)	
Bachelor’s degree	377 (36.3)	166 (35.9)	211 (35.3)	
Graduate degree	353 (33.9)	157 (34.6)	196 (33.9)	
Vocational/technical school	33 (3.2)	19 (4.2)	14 (2.5)	
Not reported	8 (0.8)	4 (0.9)	4 (0.7)	
Missing	18 (1.7)	1 (0.2)	17 (3.0)	
**Annual family income^d^, $**				
<50,000	141 (13.6)	70 (15.3)	71 (12.2)	.048
50,000–74,999	90 (8.7)	43 (9.1)	47 (8.3)	
75,000–99,999	120 (11.5)	69 (15.1)	51 (8.5)	
≥100,000	501 (48.2)	212 (46.8)	289 (50.4)	
Missing	188 (18.1)	62 (13.6)	126 (21.6)	
**Housing type^d^**				
Single family	665 (69.1)	395 (73.0)	270 (64.0)	.04
Other	286 (29.7)	137 (25.3)	149 (35.3)	
Don’t know/not sure	2 (0.2)	2 (0.4)	0 (0.0)	
No answer	10 (1.0)	7 (1.6)	3 (0.7)	
**Body mass index for age, percentile^d^**				
<5th	81 (8.0)	38 (8.4)	43 (7.6)	.33
5th to <85th	726 (71.3)	330 (73.2)	396 (69.8)	
>85th to <95th	105 (10.3)	38 (8.4)	67 (11.8)	
>95th	70 (6.9)	29 (6.4)	41 (7.2)	
Missing	36 (3.5)	16 (3.5)	20 (3.5)	

Abbreviations: BCFH, breast cancer family history; LEGACY, Lessons in Epidemiology and Genetics of Adult Cancer from Youth.

a Lessons in Epidemiology and Genetics of Adult Cancer from Youth (25).

b BCFH positive indicates girl had a family history of breast cancer in 1 or more first- or second-degree relatives. BCFH negative indicates girl had no family history of breast cancer in first- or second-degree relatives.

c Differences in age and associated *P* values were examined and calculated by using *t* tests; Differences in all other variables were examined by using χ^2^ analyses; significant at *P* < .05.

d All data are shown as n (%), unless otherwise indicated.

**Table 3:** Lifestyle Characteristics of Participants, LEGACY Girls Study^a^, by Breast Cancer Family History (BCFH) Status, 2011

Characteristic	All Girls, N = 1,040	BCFH Negative,^b^ n = 456	BCFH Positive,^b^ n = 584	*P* Value^c^
Daughters’ physical activity outside of school – sports, min/wk, mean (SD)	468.21 (1,001.22)	459.22 (876.69)	475.22 (1,089.30)	.79
Mother/guardian’s diet score^d^, n (mean) [SD]	717 (31.53) [3.83]	357 (31.58) [3.76]	360 (31.48) [3.91]	.74
Daughters’ HEI score^e^, n (mean) [SD]	961 (62.02) [9.70]	469 (61.94) [9.74]	492 (62.10) [9.67]	.80
Mothers’ physical activity (min/week), mean (SD)	206.21 (209.98)	212.87 (218.79)	201.10 (202.88)	.33
Fathers’ physical activity (min/week), mean (SD)	144.97 (254.16)	160.39 (315.12)	132.93 (193.10)	.10
Guardians’ family relationship score^e^, n (mean) [SD]	664 (2.48) [0.18]	328 (2.47) [0.01]	336 (2.42) [0.02]	.53
Daughters’ family relationship score^f^, n (mean) [SD]	335 (2.45) [0.22]	157 (2.47) [0.21]	178 (2.42) [0.24]	.10

Abbreviations: LEGACY, Lessons in Epidemiology and Genetics of Adult Cancer from Youth; HEI, Healthy Eating Index; SD, standard deviation.

a Lessons in Epidemiology and Genetics of Adult Cancer from Youth (25).

b BCFH positive indicates girl had a family history of breast cancer in 1 or more first- or second-degree relatives. BCFH negative indicates girl had no family history of breast cancer in first- or second-degree relatives. Differences in characteristics between BCFH positive and BCFH negative were examined by using *t* tests.

c *P* values calculated by *t *test; significant at *P* < .05.

d Out of 42; a higher number indicates a healthier diet score.

e Out of 100; a higher number indicates a healthier diet score.

f Out of 4; a higher relationship score indicates a healthier family relationship.

Because our analysis included regression modeling with imputed values for missing data, we compared the characteristics of girls included in the model with those of girls with missing data (Table 1). Compared with girls excluded from the regression model because of missing data, girls included had slightly higher BMI (17.60 vs 17.11 kg/m^2^, *P* = 0.02) and were nearly 1 year older (9.66 y vs 8.71 y, *P* = .004). No other characteristics differed between the groups.

**Bivariate correlations**. Bivariate correlation analysis showed a significant correlation between daughters’ HEI score and mothers’ diet score (*r*[668] = 0.25, *P* = .003), mothers’ physical activity (*r*[970] = 0.12, *P* = .002), fathers’ physical activity (*r*[970] = 0.08, *P* = .01), and family income (*r*[854] = 0.13, *P* = .006) (Table 4). Mothers’ physical activity was significantly correlated with fathers’ physical activity (*r*[1,044] = 0.13, *P* = .009), mothers’ diet score (*r*[722] = 0.29, *P* = .001), and daughters’ BMI (*r*[985] = −0.07, *P* = .03). Additionally, mothers’ diet score was significantly correlated with daughters’ BMI (*r*[683] = −0.14, *P* = .008) and family income (*r*[651] = 0.21, *P* = .001).

**Table 4:** Bivariate Correlations (Pearson *r*) Among Families’ Physical Activity, Diet, Relationships, and Demographic Characteristics, LEGACY Girls Study^a^, 2011

Variable	Daughters’ HEI Score^b^	Mothers’ diet score^c^	Daughters’ sports participation	Mothers’ Physical Activity	Fathers’ Physical Activity	Mothers’ FAD score^d^	Daughters’ BMI^e^	Daughters’ age	Daughters’ race/ethnicity
Mothers’ diet score	0.25^f^	—	—	—	—	—	—	—	—
Daughters’ sports participation	−0.01	0.03	—	—	—	—	—	—	—
Mothers’ physical activity	0.12^f^	0.29^f^	0.03	—	—	—	—	—	—
Fathers’ physical activity	0.08^f^	0.04	0.03	0.13^f^	—	—	—	—	—
Mothers’ FAD^c^ score	−0.01	0.05	−0.04	0.07	−0.05	—	—	—	—
Daughters’ BMI^d^	−0.03	−0.14^f^	−0.03	−0.07^f^	−0.01	0.02	—	—	—
Daughters’ age	−0.02	0.00	0.04	−0.02	−0.01	0.05	0.41^f^	—	—
Daughters’ race/ethnicity	0.02	0.01	−0.03	0.06^f^	0.14^f^	−0.02	0.00	0.02	—
Income	0.13^f^	0.21^f^	0.12^f^	0.04	0.09^f^	0.05	−0.02^f^	−0.02	−0.07^f^

Abbreviations: —, not applicable; BMI, body mass index; FAD, family assessment device; HEI, healthy eating index.

a Lessons in Epidemiology and Genetics of Adult Cancer from Youth (25).

b Out of 42; a higher number indicates a healthier diet score.

c Out of 100; a higher number indicates a healthier diet score.

d Out of 4; a higher relationship score indicates a healthier family relationship.

e Calculated as weight in kg/height in m^2^.

f Significant at *P* < .05.

**Regression analysis**. Before regression analysis, tests to see whether data met criteria for collinearity indicated that multicollinearity was not a concern among the pertinent predictor variables (mothers’ diet score: tolerance = 0.93, VIF = 1.08; daughters’ sports participation: tolerance = 0.99, VIF = 1.01; mothers’ physical activity: tolerance = 0.89, VIF = 1.11; fathers’ physical activity: tolerance = 0.96, VIF = 1.05; mothers’ FAD score: tolerance = 0.98, VIF = 1.02). Both mothers’ diet (β coefficient = 0.71; 95% CI, 0.46–0.88; *P* = .005) and family income (β coefficient = 3.28; 95% CI, 0.79–5.78; *P* = .002) were significantly associated with daughters’ HEI score (Table 5). For BCFH positive families, mothers’ diet score (β coefficient = 0.73; 95% CI, 0.42–1.03, *P* = .006) and family income (β coefficient = 6.24; 95% CI, 2.68–9.80; *P* = .004) were significantly associated with daughters’ HEI score (Table 6). For BCFH negative families, only mothers’ diet score (β coefficient = 0.62; 95% CI, 0.29–0.95; *P* = .001) was significantly associated with daughters’ HEI score. In the model that used imputed values the results were very similar, with only mothers’ diet and family income being predictive of daughters’ HEI.

**Table 5:** Parameter Estimates From the Mixed Effects Linear Regression Model Predicting the Outcome of Daughter’s Healthy Eating Index (HEI) Score, LEGACY Girls Study^a^, 2011

Predictors	Model 1^b^, n = 602	Model 2^c^, n = 522
**Mothers’ diet score^d^**	0.63 (0.43 to 0.83)^e^	0.71 (0.46 to 0.88)^e^
**Daughters’ sports participation**	0.001 (−0.001 to 0.001)	−0.001 (−0.001 to 0.001)
**Mothers’ physical activity**	0.001 (−0.003 to 0.005)	0.001 (−0.004 to 0.004)
**Fathers’ physical activity**	0.003 (−0.001 to 0.007)	0.003 (−0.001 to 0.007)
**Mothers’ FAD score^f^**	−0.08 (−0.43 to 0.27)	−0.15 (−0.54 to 0.23)
**Daughters’ body mass index^g^**	—	0.20 (−0.04 to 0.45)
**Daughters’ age**	—	−0.12 (−0.49 to –0.26)
**Daughters’ race/ethnicity**		
Hispanic White	—	−2.51 (–4.23 to 1.89)
Hispanic Black	—	−0.59 (–1.98 to 2.04)
White	1 [Reference]	
African American/Black	—	−2.72 (–3.42 to 2.18)
Native Hawaiian/Other Pacific Islander	—	−0.52 (–1.22 to 3.12)
Asian	—	−0.52 (–1.35 to 2.85)
Mixed race/ethnicity	—	−0.62 (–2.32 to 3.20)
**Annual family income, $**		
<50,000	1 [Reference]	
50,000–74,999	—	0.43 (−2.86 to 3.71)
75,000–99,999	—	0.04 (−3.02 to 3.09)
≥100,000	—	3.28 (0.79 to 5.78)^e^

Abbreviations: —, not applicable; FAD, family assessment device.

a Lessons in Epidemiology and Genetics of Adult Cancer from Youth (25). Values are β coefficient (95% CI).

b Base model; includes only lifestyle predictors: mothers’ and fathers’ physical activity, family relationship scores, mothers’ diets, and daughters’ sports participation.

c Contained all variables from Model 1 with the addition of select covariates: daughters’ age and race/ethnicity, and family income.

d Out of 42; a higher number indicates a healthier diet score.

e Significant at *P* < .05.

f Out of 4; a higher relationship score indicates a healthier family relationship.

g Calculated as weight in kg/height in m^2^.

**Table 6:** Parameter Estimates From Mixed-Effects Linear Regression Models Predicting the Outcome of Daughter’s Healthy Eating Index (HEI) Score by Breast Cancer Family History Status (BCFH), LEGACY Girls Study^a^, 2011

Predictors	BCFH Positive		BCFH Negative	
	Model 1^b^, n = 301	Model 2^c^, n = 269	Model 1^b^, n = 298	Model 2^c^, n = 250
**Mothers’ diet score**	0.77 (0.48 to 1.06)^d^	0.73 (0.42 to 1.03)^d^	0.71 (0.42 to 1.05)^d^	0.62 (0.29 to 0.95)^d^
**Daughters’ sports participation**	0.001 (−0.002 to 0.002)	−0.001 (−0.001 to –0.001)	0.001 (−0.001 to 0.001)	−0.001 (−0.002 to 0.001)
**Mothers’ physical activity**	−0.001 (−0.01 to –0.004)	0.001 (−0.006 to 0.01)	0.003 (−0.003 to 0.01)	−0.001 (−0.01 to 0.01)
**Fathers’ physical activity**	0.003 (−0.001 to 0.01)	0.006 (−0.001 to 0.13)	0.003 (−0.003 to 0.01)	0.001 (−0.004 to 0.01)
**Mothers’ FAD score^c^**	−0.14 (−0.67 to –0.39)	−0.09 (−0.66 to 0.47)	−0.05 (−0.55 to 0.44)	−0.16 (−0.69 to 0.38)
**Daughters’ BMI^d^**	^—^	0.21 (−0.14 to 0.55)	^—^	0.09 (−0.25 to 0.43)
**Daughters’ age**	^—^	−0.31 (−0.85 to 0.24)	^—^	0.06 (−0.46 to 0.58)
**Daughters’ race/ethnicity**				
Hispanic White	^—^	−5.87 (−13.79 to 2.05)	^—^	0.57 (−9.66 to 10.81)
Hispanic Black	^—^	−2.95 (−6.51 to 0.60)	^—^	1.42 (−1.98 to 4.82)
White	1 [Reference]			
African American/Black	^—^	−3.76 (−8.20 to 0.67)	^—^	−1.94 (−8.33 to 4.45)
Native Hawaiian/other Pacific Islander	^—^	−3.93 (−8.79 to 0.93)	^—^	2.31 (−2.56 to 7.18)
Asian	^—^	−9.21 (−27.39 to 8.97)	^—^	2.27 (−5.19 to 9.73)
Mixed race/ethnicity	^—^	−1.003 (−8.13 to 6.12)	^—^	−1.14 (−9.13 to 7.15)
**Annual income, $**				
<50,000	1 [Reference]			
50,000–74,999	^—^	1.02 (−3.53 to 5.56)		−0.44 (−5.03 to 4.42)
75,000–99,999	^—^	2.53 (−1.69 to 6.76)		−2.76 (−7.18– to 1.67)
≥100,000	^—^	6.24 (2.68 to 9.80)^d^		0.36 (−3.28 to 4.00)

Abbreviations: —, not included in the model; BCFH, breast cancer family history; BMI, body mass index; FAD, Family Assessment Device.

a Values are β coefficient (95% CI).

b Base model (Model 1) includes only lifestyle predictors: mothers’ and fathers’ physical activity, family relationship scores, mothers’ diets, and daughters’ sports participation.

c All variables from Model 1 with the addition of select covariates: daughters’ age and race/ethnicity, and family income.

d Significant at *P* < .05.

## Discussion

HEI for all daughters in our study was significantly and positively correlated with their mothers’ diet, mother and fathers’ physical activity, and family income, although all correlations were weak. Regression analysis showed that mothers’ diet and family income were significantly and positively associated with HEI for all participating daughters. These findings are important in the context of American HEI norms, which indicate poor adherence to national dietary guidelines, with an average score of 53.0 out of a maximum 100.0 for children aged 6 to 17 years and an average score of 59.0 for adults. For context, the daughters in our study had an average HEI score of 62.0, which did not differ significantly between BCFH positive (62.1) and BCFH negative (61.9) groups. That HEI is linked to family income is unsurprising. Foods with higher nutritional quality are more expensive per calorie than those with lower value, and low-income groups often choose foods that are less nutrient dense because they cost less (34). Overall, the higher the family income, the greater the ability to provide diverse and healthy food. Because our sample included families aware of their history of breast cancer, we were able to assess whether this awareness resulted in differences in family behaviors, such as diet and physical activity, which are modifiable risk factors for breast cancer. We found little difference between BCFH positive and BCFH negative families for daughters’ BMI, HEI score, or total physical activity; for mother/guardians’ diet score or physical activity of the mother or father; or the family relationship score reported by girls or mothers.

Because 71% of US mothers, compared with 18% of fathers, are their family’s primary food shoppers and preparers, these activities could account for the consistent association between mothers’ diet and daughters’ HEI (35). In addition, if the mother is the food shopper and preparer, she also models meal development and eating behaviors for her daughter, whether the family is BCFH positive or negative. Parental food access and behavior modeling are strongly associated with children’s food choices (36). When mothers emphasize health goals for their family, children consume more healthful and less unhealthful food (37). Children also may influence food choice when shopping with parents (38), so mother and daughter can be considered a dyad, each having some influence on the decision to eat healthfully, an important consideration for BCFH positive families, wherein the mother’s diet is associated with her daughter’s HEI. Given the low HEI scores for both mothers and daughters in our study and the association of dietary components with breast cancer risk, further work is needed to identify effective communication strategies and behavioral interventions for BCFH positive families. Such interventions should use prior knowledge about parental modeling and the unique dynamics of the mother–daughter relationship. Previous research and interventions focused on adopting healthy behaviors for the whole family; however, our study results suggest that future research should determine whether strategies targeted at the mother and daughter dyad are more effective.

In addition, family income has been positively linked to physical activity and sports participation for young people in middle- and high-income families. Vella et al (39) found that at age 8, a high household income predicted participation in sports, whereas low income predicted dropout from organized sports. Others found that low family income was associated with poor fitness and high risk of obesity for children (40). Researchers examining barriers to physical activity in low-income communities in Colorado found the most commonly cited barriers were lack of low-cost options and traffic safety (41). We also found that daughter’s sports participation and family income were significantly and positively correlated.

Our findings that both mother’s and father’s self-reported physical activity significantly and positively correlated with daughter’s HEI score highlight the importance of viewing children’s health behaviors from a family perspective. Other studies used this family approach by studying mother–father–child triads and found that multiple health behaviors, including physical activity, healthy food consumption, and less sedentary time, co-occurred at the family level (19). Although many health behavior interventions have been with mothers and daughters (20), less work has been done with fathers and daughters. Future work should continue to consider the father’s influence on a daughter’s health behaviors.

Separate regression analyses for BCFH negative and BCFH positive families showed that the mother’s diet score was the only significant predictor of the daughter’s HEI for BCFH negative families, whereas for BCFH positive families, both the mother’s diet score and family income significantly predicted the daughter’s HEI. These findings further emphasize the importance of the mother–daughter relationship when considering how daughters might be encouraged to establish healthy eating habits, regardless of a family history of breast cancer. Although our study found that family income significantly predicted the daughter’s HEI only in BCFH positive families, further studies should explore how family income might play a role in BCFH negative families and/or families with a history of other types of cancers.

A strength of our study was the inclusion of girls from both BCFH negative and positive families, which is representative of the general US population. Thus, our findings can be used to better understand the many issues around healthy eating, diet, and physical activity for girls from both types of families.

Our study had limitations. First, all measures except BMI were self-reported, which introduced bias and a certain degree of measurement error. Second, a large amount of missing data in the LEGACY baseline questionnaires resulted in the loss of roughly half of the observations from our regression model. As a result, we also ran the regression model with multiple imputation, and found no substantive differences between the 2 models. However, we did not identify a strong variable on which to base the imputation. As such, the model with multiple imputation may represent an imperfect imputation rather than a finding that imputation made no difference in the regression results. Nevertheless, the comparison of characteristics reported on girls included and excluded from the model did not show important differences between groups, suggesting that the model without imputation was probably representative of the larger study sample. Additionally, because all measures except for BMI were self-reported, this introduced bias and a certain degree of measurement error; therefore, mothers’ dietary data were derived from 7 questions from a validated tool rather than a complete food frequency questionnaire. Finally, because our study was cross-sectional, causation between any of the variables cannot be assumed.

Parents’ behaviors are important in forming girls’ eating habits, and lifestyle changes are best addressed in a family context. This is particularly true for mothers’ dietary behaviors, probably because mothers are most involved in food shopping and preparation. However, recent literature suggests that fathers play some role in their daughters’ eating habits, weight, and body image (42,43), and more research is needed in this area, particularly in light of evolving family roles. Finally, health interventions aimed at reducing breast cancer risk in BCFH positive families could be part of other lifestyle interventions for young people that focus on improving eating habits. Improving eating habits in BCFH positive families could reduce breast cancer risk; however, further study is warranted. Overall, our findings reinforce the importance of parental influence on daughters’ eating habits and the value of a family-focused approach to addressing breast cancer prevention and overall health.
